# Preparation of g-C_3_N_4_/Graphene Composite for Detecting NO_2_ at Room Temperature

**DOI:** 10.3390/nano7010012

**Published:** 2017-01-12

**Authors:** Shaolin Zhang, Nguyen Thuy Hang, Zhijun Zhang, Hongyan Yue, Woochul Yang

**Affiliations:** 1 Department of Physics, Dongguk University, Seoul 04620, Korea; slzhang@dongguk.edu (S.Z.); thuyhang.clc@gmail.com (N.T.H.); zjzhang@dongguk.edu (Z.Z.); 2 School of Materials Science and Engineering, Harbin University of Science and Technology, Harbin 150040, China; hyyue@163.com

**Keywords:** graphene, g-C_3_N_4_, composite, gas sensor, room temperature

## Abstract

Graphitic carbon nitride (g-C_3_N_4_) nanosheets were exfoliated from bulk g-C_3_N_4_ and utilized to improve the sensing performance of a pure graphene sensor for the first time. The role of hydrochloric acid treatment on the exfoliation result was carefully examined. The exfoliated products were characterized by X-ray diffraction (XRD) patterns, scanning electron microscopy (SEM), atomic force microscopy (AFM), and UV-Vis spectroscopy. The exfoliated g-C_3_N_4_ nanosheets exhibited a uniform thickness of about 3–5 nm and a lateral size of about 1–2 µm. A g-C_3_N_4_/graphene nanocomposite was prepared via a self-assembly process and was demonstrated to be a promising sensing material for detecting nitrogen dioxide gas at room temperature. The nanocomposite sensor exhibited better recovery as well as two-times the response compared to pure graphene sensor. The detailed sensing mechanism was then proposed.

## 1. Introduction

Gas sensors are devices that able to respond to specific gasses and they play an important role in industrial chemical processing, environmental monitoring, agriculture, medicine, public safety, and indoor air quality control [1]. Up to now, most commercial gas sensors have been based on metal oxide semiconductors due to their numerous advantages such as low cost, simplicity in measurements, high sensitivity towards various gases with ease of fabrication, and high compatibility with other processes [2,3,4]. However, these conventional sensors generally require high working temperatures (200 °C to 500 °C); this not only degrades the long-term sensing performance but also greatly limits their applications, e.g., wearable devices [5,6,7].

Recent studies have revealed that two-dimensional (2D) materials, especially graphene, have the potential to detect numerous gases at room temperature owing to their large surface area, high carrier mobility, and low noise. However, many reports have also demonstrated that pristine graphene has a poor gas sensing property owing to the absence of dangling bonds in the structure [8,9]. In order to solve this problem, a doping process and defect engineering have been conducted to improve the gas adsorption of graphene [10,11]. On the other hand, compositing methods such as decorating graphene with metal and metal oxide nanoparticles, as well as with polymers, have also been studied. Compared to pristine graphene, sensors based on graphene compositing with nanoparticles of metals or metal oxides have demonstrated highly sensitive and selective sensing behavior [12,13,14,15], which could be attributed to the excellent catalytic properties and synergistic effects of the partner materials [16,17,18].

On the other hand, graphitic carbon nitride (g-C_3_N_4_), as another emerging component with a two-dimensional characteristic structure has attracted considerable attention recently, due to the tri-s-triazine units that are connected by amino groups in each layer and the weak van der Waals forces between layers. The presence of nitrogen atoms in the graphene-like layered g-C_3_N_4_ structure gives g-C_3_N_4_ its unique properties, such as semiconductor properties, solid alkalinity, and complexing ability, thus endowing it with better performance than graphene in some aspects [19,20]. g-C_3_N_4_ has been extensively used as a polymeric photo-catalyst for solar hydrogen production and environmental purification, as well as oxygen reduction and evolution [21,22,23]. A few recent studies have also reported that nanostructured g-C_3_N_4_ exhibits excellent gas sensing properties owing to its excellent catalytic property [24,25]. Considering the similar 2D structures as well as the complementary properties between g-C_3_N_4_ and graphene, a synergetic effect in sensing performance could be expected if the two components are composited as a gas sensor material.

In this study, we investigated the gas sensing performance of the g-C_3_N_4_/graphene composite at room temperature. g-C_3_N_4_ nanosheets were prepared using an acid treatment enhanced liquid-phase exfoliation process. The effect of acid treatment time on exfoliation was carefully investigated. The obtained g-C_3_N_4_ nanosheets were mixed with exfoliated graphene nanosheets to form a composite which was then tested with NO_2_. The results demonstrated that compositing g-C_3_N_4_ with graphene greatly improves the sensing performance of pure graphene. The sensing mechanism was carefully discussed.

## 2. Results and Discussion

### 2.1. Acid Treatment Enhanced Exfoliation

**Figure 1:**
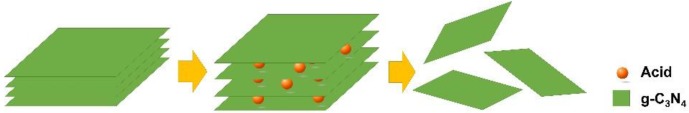
Schematic of the acid treatment enhanced liquid-phase exfoliation process from bulk g-C_3_N_4_ to ultrathin nanosheets.

Up to now, the lack of a simple approach to producing 2D nanomaterials at a large scale has been the main obstacle to achieving wide application of 2D graphene-like materials. The feasibility of the recently developed ultrasound-assisted liquid-phase exfoliation method has been demonstrated for producing ultrathin nanosheets from bulk 2D material. The cavitation effect induced by the collapse of ultrasonic-produced bubbles generates liquid jets with high temperature, pressure, and speed, which would overcome the interlayer van der Waals force and break up the layered structure to yield individual nanosheets. However, this method is still not robust enough owing to the low yield and re-aggregation tendency after exfoliation. To address these issues, a possible route is the optimization of the solvent. It is reported that the maximization of exfoliation efficiency is related to the surface energy of the solvent. The comparative surface energy between the solvent and the 2D structured solute is favorable for the delamination. Xie and her co-researchers revealed that the surface energy of water matches well with that of g-C_3_N_4_. Their works demonstrated that the high dispersion of g-C_3_N_4_ individual layers can be realized in water [26]. Another alternative strategy is chemical modification, which involves redox reactions, intercalation, or an ion-exchange process. Typically, as shown in Figure 1, the intercalation of foreign molecules into the interlayer gallery of g-C_3_N_4_ would cause the interplanar space to swell, weakening the interlayer reaction, and thus facilitating the exfoliation. In several previous studies, sulphuric acid (H_2_SO_4_) or nitric acid (HNO_3_) was adopted to intercalate the acid ions into the interlayer space of g-C_3_N_4_ followed by an ultrasonic treatment, and ultrathin g-C_3_N_4_ nanosheets were successfully synthesized [27,28]. However, these strong oxidants might incur structural damage to the products. Mild non-oxidant hydrochloric acid (HCl) would be more suitable for preserving the intrinsic structure of g-C_3_N_4_. Moreover, in these previous studies, the controlling factors in the exfoliation process, such as acid treatment time, were rarely considered.

**Figure 2:**
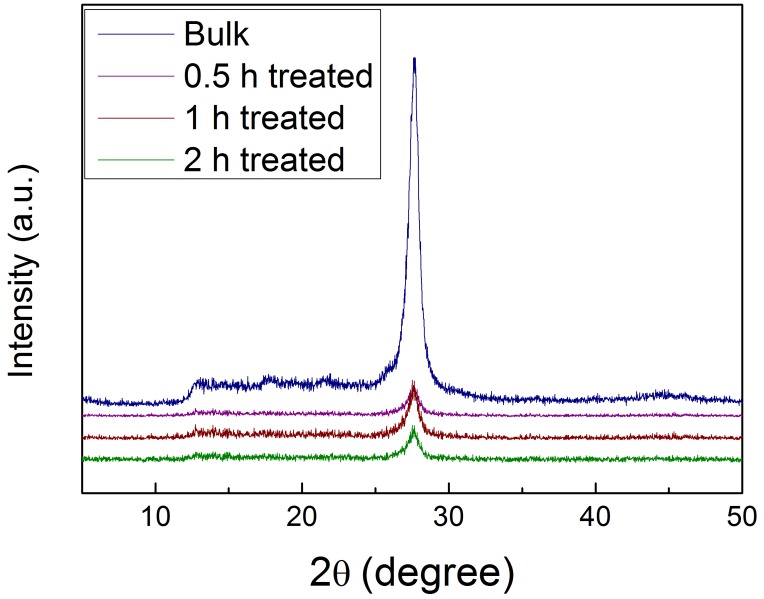
X-ray diffraction (XRD) patterns of bulk g-C_3_N_4_ and g-C_3_N_4_ nanosheets exfoliated at different acid treatment times.

The crystal structures of bulk g-C_3_N_4_ and g-C_3_N_4_ products exfoliated with different HCl acid treatment times were examined by X-ray diffraction (XRD) patterns. As shown in Figure 2, after exfoliation, both bulk g-C_3_N_4_ and the resultant g-C_3_N_4_ products remain substantially the same except for some minor changes. No obvious difference was observed between the exfoliated products. All the samples presented a characteristic peak near 27.7°, which can be indexed to the (002) facet caused by the interlayer stacking reflection of conjugated aromatic systems. It was found that the (002) peaks of exfoliated g-C_3_N_4_ presented a slight left shift from 27.7° to 27.5°, suggesting loose packing in the exfoliated g-C_3_N_4_ product. It is worth noting that the intensity of the (002) peak decreased significantly in the exfoliated sample compared with the bulk sample, demonstrating the successful exfoliation of bulk g-C_3_N_4_. It was also observed that the sensitivity of the diffraction peak at 12.74°, which represents the period tri-s-triazine group, was reduced in the exfoliated g-C_3_N_4_ compared with the bulk g-C_3_N_4_. This could be attributed to the downgrade of the planar size after exfoliation.

**Figure 3:**
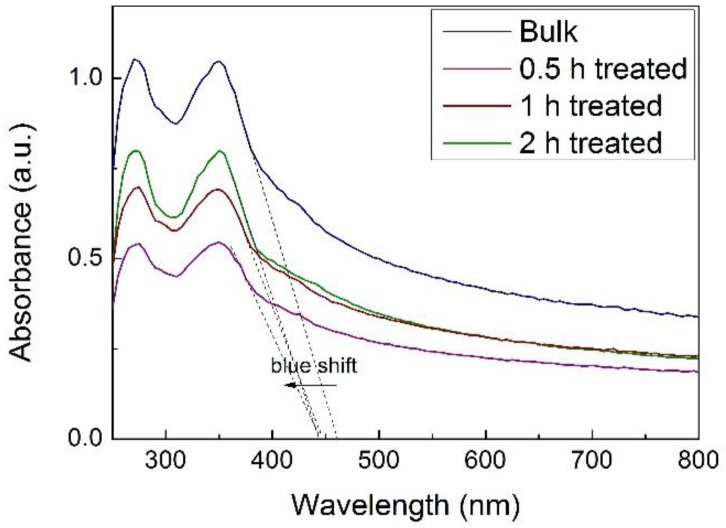
UV-Vis absorption spectra of diluted bulk g-C_3_N_4_ and as prepared g-C_3_N_4_ nanosheets exfoliated at different acid treatment times.

The light absorbance of the samples was studied by UV-Vis spectroscopy. As shown in Figure 3, g-C_3_N_4_ displayed photoabsorption from ultraviolet to visible light. The bulk g-C_3_N_4_ has a band edge at approximately 460 nm corresponding to 2.70 eV. For comparison, all the exfoliated g-C_3_N_4_ samples have a band edge at approximately 450 nm, corresponding to a bandgap of 2.74 eV, indicating a slight blue shift of 0.04 eV. The increase of bandgap after exfoliation can be attributed to the well-known quantum confinement effect. When the thickness of the g-C_3_N_4_ product after exfoliation becomes ultrathin, approaching the dimension of the de Broglie wavelength, the motion of electrons is confined, leading to the splitting of energy levels, and thus the conduction and valence bands shift in opposite directions, resulting in the increase of the bandgap energy.

The UV-Vis absorption spectra of the samples also provide information of the concentration of the g-C_3_N_4_ products. According to the Lambert-Beer law of *A*/*l* = α*C*, where α is absorption coefficient, *A* is the absorbance, *l* is light path length, and *C* is the concentration of solute, the absorption of light is directly proportional to both the concentration of the solute and the thickness of the medium in the light path. Given that the light path was fixed throughout the measurement, the concentration of g-C_3_N_4_ could be estimated from the intensity of absorption. As observed from Figure 3, the g-C_3_N_4_ product with 0.5 h acid treatment exhibited the lowest concentration. The concentration increased considerably as the acid treatment time extended to 1 h, and then slightly increased as the time further increased to 2 h, suggesting the exfoliation approached saturation.

**Figure 4:**
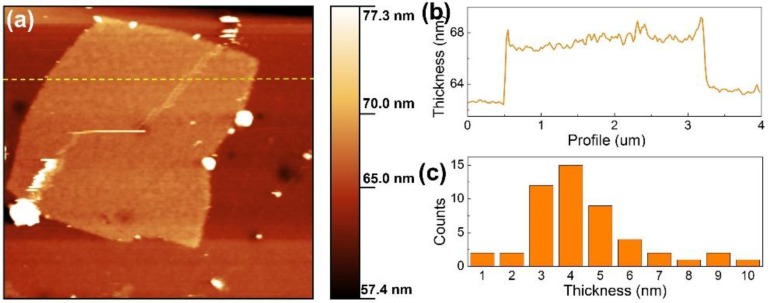
(**a**) Typical atomic force microscopy (AFM) image; (**b**) corresponding thickness profile along the yellow dashed line in (**a**), and (**c**) thickness distribution of the exfoliated g-C_3_N_4_ with 1 h acid treatment.

The morphology of the exfoliated g-C_3_N_4_ with 1 h HCl acid treatment was investigated using atomic force microscopy (AFM). As shown in Figure 4a, a well-defined sheet-like g-C_3_N_4_ nanostructure was observed. The thickness of the g-C_3_N_4_ nanosheet was estimated to be about 4 nm (Figure 4b). Considering the theoretical thickness of monolayer g-C_3_N_4_ is about 0.3–0.4 nm [20], this AFM result suggests the obtained g-C_3_N_4_ nanosheet comprises about 10 layers. A total of more than 50 AFM samples have been examined and the statistical thickness distribution is shown in Figure 4c. The figure shows that, for over 70% of the product, the thickness ranges from 3 to 5 nm.

**Figure 5:**
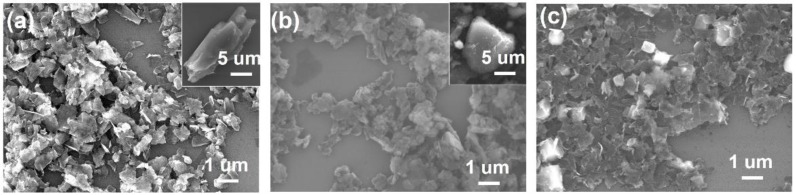
Field emission scanning electron microscopy (FE-SEM) images of exfoliated (**a**) graphene; (**b**) g-C_3_N_4_; and (**c**) g-C_3_N_4_/graphene composite. The insets in (**a**,**b**) are SEM images of their bulk counterparts.

The microstructures of the graphene and g-C_3_N_4_ nanosheets, and their composite were investigated through field emission scanning electron microscopy (FE-SEM). Figure 5a,b presents the SEM images of exfoliated graphene and g-C_3_N_4_, respectively. A large number of loosely stacked graphene nanosheets could be observed, suggesting successful exfoliation. On the other hand, the exfoliated g-C_3_N_4_ nanosheets exhibited a severe restacking, whereby it is difficult to identify an individual nanosheet. In the case of the composite, as shown in Figure 5c, porous g-C_3_N_4_/graphene film was observed. Remarkably, the restacking phenomenon of g-C_3_N_4_ was greatly alleviated, which could be attributed to the self-assembly process between g-C_3_N_4_ and graphene [23]. This finding reveals that compositing 2D materials would be favorable in preventing the restacking of the components. It was noted that the evidence of compositing is difficult find even by high-resolution SEM (Figure S1), owing to the resemblance of 2D nanomaterials. Other characterization methods would be required to further examine the g-C_3_N_4_/graphene composite.

**Figure 6:**
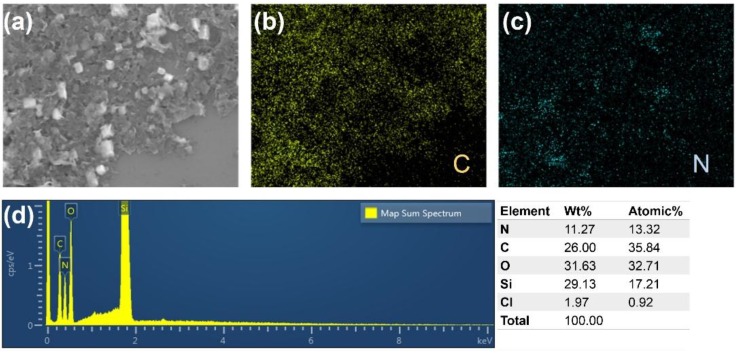
(**a**) Low-magnification SEM image of g-C_3_N_4_/graphene nanocomposite; (**b**) Carbon and (**c**) nitrogen elemental mapping captured in (**a**); (**d**) Energy-dispersive X-ray spectroscopy (EDS) pattern and elemental composition of nanocomposite.

Energy-dispersive X-ray spectroscopy (EDS) analysis and elemental mapping were also utilized to investigate the elemental composition and distribution of the g-C_3_N_4_/graphene composite, respectively. The elemental mapping analysis, as shown in Figure 6a–c, shows that the C and N elements are homogeneously dispersed, indicating the successful formation of the nanocomposite. According to the EDS spectrum in Figure 6d, besides the Si and O elements contributed by the oxidized silicon wafer, the main elements of the nanocomposite were N and C. The atomic ratio of both N and C was about 1:3, which is consistent with the starting ratio. Besides, it is noted that a neglectable amount of Cl element was observed in the composite. This was originated from the strongly bonded Cl on the defective sites of g-C_3_N_4_ [29].

X-ray photoelectron spectroscopy (XPS) measurement was carried out to further study the chemical composition and element binding energies of g-C_3_N_4_/graphene nanocomposite, as displayed in Figure S2. The survey XPS spectra (Figure S2a) obviously indicated the co-existence of the elements C, N, and a small amount of O. No Cl component was observed, suggesting that most of the Cl ions were removed in the washing process. The emergence of element O may be attributed to the adventitiously adsorbed contaminant, such as surface adsorbed H_2_O [30,31,32]. The C1s spectrum displayed in Figure S2b can be deconvoluted into three peaks with binding energies of 288.2, 286, and 284.7 eV, which are assigned to N–C=N, N–C, and sp^2^ C=C, respectively [24,29]. No C–O or C=O bond were observed revealing that the graphene preserved its structure and no oxidation occurred throughout the process. On the other hand, the N1s XPS spectra (Figure S2b) displayed two peaks at 400.3 and 398.7 eV, which are assigned to C–N and C=N–C, respectively. The C=N–C represents the sp^2^ hybridized aromatic N bonded to carbon atoms (C=N–C) existed in g-C_3_N_4_, which was known as the reactive pyridine N. Besides, the surface N/C ratio in nanocomposite was determined to be 0.27, which is closed to that in EDS analysis. The above XPS results further demonstrated the formation of g-C_3_N_4_/graphene nanocomposite.

### 2.2. Gas Sensing Performance

**Figure 7:**
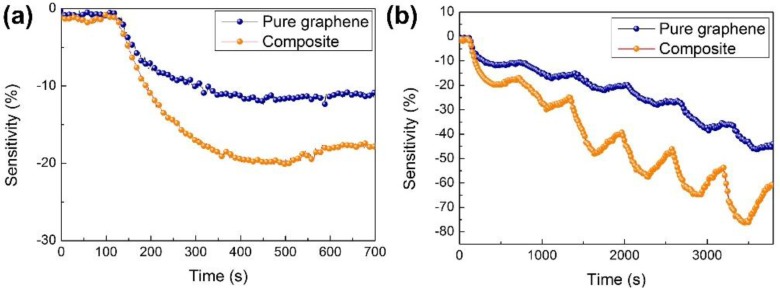
(**a**) Typical response of pure graphene and g-C_3_N_4_/graphene composite sensors toward 5 ppm of NO_2_ gas at room temperature; (**b**) Dynamic response of pure graphene and g-C_3_N_4_/graphene composite sensors toward various concentrations of NO_2_ gas at room temperature.

The performance of the g-C_3_N_4_/graphene composite gas sensor was examined with NO_2_ gas. The pure graphene sensor as a control sample was also tested simultaneously. Figure 7a shows the typical response curve of the sensors upon exposure to 5 ppm of NO_2_ at room temperature. As observed, upon exposure to NO_2_ gas, the resistance of both sensors decreased, suggesting a p-type behavior. It is known that NO_2_ as a typical electron withdrawer would increase the hole concentration of the sensor materials, and thus decrease (or increase) the resistance of *p*-type (or *n*-type) semiconductors. Remarkably, the composite sensor exhibited two-times the response of pure graphene, indicating that the incorporation of g-C_3_N_4_ would benefit the sensing performance of the graphene sensor.

It is well-known that intrinsic graphene shows poor recovery, partially due to the strong adsorption energy of the gas molecule. Thus, heating at an elevated temperature or vacuum treatments are required to clean the graphene sensor [33,34,35]. As observed in Figure 7a, it is noted that the pure graphene sensor barely showed recovery when the NO_2_ gas was removed, while about 20% recovery was observed in the composite sensor within 200 s. This is possibly attributed to the thermal excitation of g-C_3_N_4_ that introduced active carriers and facilitated the desorption process of NO_2_ gas molecules. Our results indicate that the incorporation of specific semiconductors can achieve full recovery of the graphene sensor without the need for a complicated heating or vacuum process.

**Figure 8:**
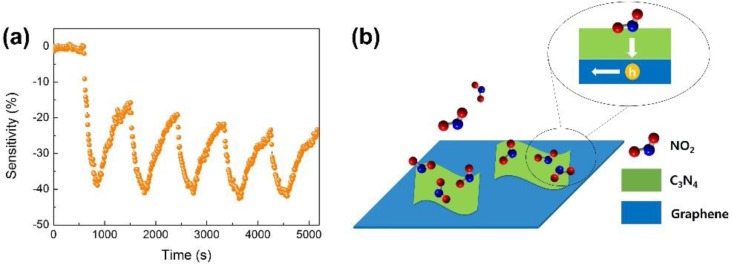
(**a**) Cyclic response of g-C_3_N_4_/graphene composite sensor toward 20 ppm of NO_2_; (**b**) Proposed sensing mechanism of g-C_3_N_4_/graphene composite sensor.

Figure 7b shows the dynamic response of pure graphene and g-C_3_N_4_/graphene composite sensors toward NO_2_ ranging from 5 to 200 ppm. The sensors responded well with the target gas. The responses increased monodirectionally as the concentration of NO_2_ increased. No obvious saturation state was observed in the composite sensor, suggesting a broad detection range. Remarkably, the composite sensor showed two-times more response than the other sensors to pure graphene in all the tests. The stability property of our nanocomposite sensor was also examined as shown in Figure 8a. The nanocomposite sensor responded repeatedly upon cycled exposure to 20 ppm of NO_2_, suggesting an excellent repeatability.

The sensing mechanism of the g-C_3_N_4_/graphene composite sensor is proposed, as schematically shown Figure 8b. On the one hand, the triazine structure of g-C_3_N_4_ nanosheet behaves similarly to a base that is ready to interact with the oxidized NO_2_ gas molecules. This characteristic of g-C_3_N_4_ nanosheet as well as its excellent catalytic property endows it with a promising capacity for the adsorption of NO_2_ molecules. On the other hand, graphene with superior carrier mobility would act similarly to a signal highway in the sensing process. Upon adsorption, the oxidized NO_2_ gas molecule with strong electron affinity deposits a hole in the g-C_3_N_4_ nanosheet. These hole carriers are then rapidly conducted by the graphene. In short, the complementary natures of g-C_3_N_4_ and graphene in the sensing process introduce a synergetic effect that contributes to the excellent sensing property of the nanocomposite sensor. In addition, considering the different roles of both materials in the sensing process, a tradeoff on the sensing performance would be expected if the composition of the composite is changed. Through systematic investigation, an optimized ratio of g-C_3_N_4_ and graphene could be achieved to obtain a high-performance sensor with excellent sensitivity and promising recovery property. This is the expected direction in the future research.

## 3. Materials and Methods 

### 3.1. Preparation of Bulk g-C_3_N_4_

Raw melamine purchased from Sigma-Aldrich Korea Ltd. (Yongin, Korea) was used as received. The fabrication process was modified from the reported methods [36,37,38]. Typically, a certain amount of melamine was placed into a crucible with a cover and heated to 600 °C with a heating rate of 1 °C/min in air, and then maintained for 4 h. After natural cooling to ambient temperature, a yellow product was obtained. The obtained yellow product was subsequently ground thoroughly for further processing and characterization.

### 3.2. Acid Treatment Enhanced Exfoliation of g-C_3_N_4_

In a typical synthesis, 0.5 g of yellow bulk g-C_3_N_4_ powder was added to 25 mL hydrochloric acid (HCl, 36.46%, Deajung Korea Ltd., Siheum, Korea) and stirred for 1 h. The obtained transparent yellow dispersion was then filtrated and washed repeatedly with water until the pH value became neutral, followed by a drying process at 100 °C for 6 h. This acid-treated powder was re-dispersed in 200 mL deionized (DI) water, followed by sonication treatment for 2 h in an ultrasonic bath (200 W, NXPC-2010, Sonics & Materials, Inc., Newtown, CT, USA). Subsequently, the mixture was centrifuged at 8000 rpm for 10 min to remove the residual un-exfoliated g-C_3_N_4_ particles. Finally, a light white suspension was obtained. In order to determine the concentration of the product, 100 mL of g-C_3_N_4_ dispersion was transferred to a pre-weighted vial and dried in an oven at 100 °C for 24 h. The concentration of g-C_3_N_4_ in dispersion after 1 h acid treatment was determined to be about 50 μg/mL.

### 3.3. Preparation of Graphene 

Graphene was prepared following the process developed in our previous work [39]. Briefly, 4 g of graphite powder (Sigma-Aldrich Korea Ltd., Yongin, Korea, 20 µm) was dispersed in 200 mL of an aqueous mixture containing 20 vol % acetone and 68 vol % tetrahydrofuran. The dispersion was then ultrasonically treated for 1 h using a horn probe sonic tip (Sonic VCX 750, Sonics & Materials, Inc., Newtown, CT, USA). A cooling water system was used to keep the processing temperature below 5 °C throughout. The ultrasonic power was set to 600 W with a pulse for 20 s on and 10 s off. After the ultrasonic treatment, the black dispersion was centrifuged at 1500 rpm for 30 min. The supernatant was then carefully decanted and retained for the next process. The concentration of the graphene was estimated to be around 250 µg/mL.

### 3.4. Fabrication of g-C_3_N_4_/Graphene Composite Sensor

Two as-prepared dispersions containing equivalent weights of g-C_3_N_4_ and graphene were mixed with ultrasonic treatment for 1 h to achieve complete mixing. After mixing, the mixture was evaporated in an oven for 24 h at a moderate temperature (~60 °C) to improve the concentration of solute without introducing severe aggregation. Alumina substrates (4 × 4 mm) with interdigital Pt electrodes were carefully cleaned and placed on a hot-plate with a temperature of about 100 °C. Concentrated solution was then drop-casted onto the sensor substrates using micro-pipettes. The thickness of the thin films was about 1 µm and was controlled by the volume of the solution in the micro-pipettes. After coating, the alumina substrates were heated at 100 °C in an oven for 1 h to eliminate the remaining solvent and then sintered at 200 °C in Ar gas for 1 h to improve the adhesion and contact. Pure graphene-based sensor as the control sample was also prepared using the same method.

### 3.5. Measurement of g-C_3_N_4_/Graphene Composite Sensor

The sensors were placed in a stainless chamber having a total volume of 10 cm^3^. Nitrogen gas was used as the carrier gas. NO_2_ gas with a starting concentration of 500 ± 1 ppm in nitrogen was used as the target gas. The accurate concentration control of the target gas was achieved by using a mixing system equipped with mass flow controllers (MFC, Tylan 2900, Mykrolis Corporation, Billerica, MA, USA) and mass flow meters. All the measurements were conducted under ambient condition. The total gas flow of the carrier and target gases was kept at 250 sccm throughout the measurement process. The electrical conductance signal of the sensors was collected and recorded by data acquisition (Agilent 34970A, Keysight Technologies, Santa Rosa, CA, USA) through a customized clamp and wire connector. The sensing response was defined as:

$$S\left(\%\right)=\frac{R_{G}-R_{N}}{R_{N}}\times 100,$$

where *R_N_* and *R_G_* represented the resistance of the sensors upon exposure to nitrogen and target gas, respectively.

### 3.6. Material Characterization

For material characterization, samples were prepared by drop-casting the solution containing g-C_3_N_4_ or g-C_3_N_4_/graphene nanocomposite onto an oxidized silicon wafer, followed by a drying process at 80 °C for 12 h. X-ray diffraction (XRD) was performed using a Rigaku RINT2200 X-ray diffractometer (Rigaku Corporation, Tokyo, Japan) with monochromatized Cu-Kα radiation. Atomic force microscopy (AFM) measurements were carried out on a Bruker MutiMode-8 system (Bruker Corporation, Billerica, MA, USA). The microstructure, morphology, and elemental distribution of the samples were systematically investigated by Raman spectroscopy (Horiba LabRAM HR Evolution systems, Horiba, Ltd., Kyoto, Japan, operating at a wavelength of 785 nm), Scanning Electron Microscopy (SEM, JSM-7100FA, Hitachi, Ltd., Tokyo, Japan), and Energy-dispersive X-ray spectroscopy (EDS) mapping (co-equipped with SEM) (Hitachi, Ltd., Tokyo, Japan), respectively. Chemical compositions and element binding energies were analyzed using X-ray photoelectron spectroscopy (XPS) on an Ulvac-phi Veresprobe II system (Ulvac-Phi, Inc., Chigasaki, Japan) with monochromatic Al Kα as an excitation source. The absorption spectroscopy measurements were performed using a Varian Cary 6000i and a 1 cm cuvette.

## 4. Conclusions 

In summary, we successfully prepared g-C_3_N_4_ nanosheets from bulk g-C_3_N_4_ using a facile HCl acid treatment followed by an ultrasonic process. The effect of acid treatment on the exfoliation result was carefully examined. The exfoliated g-C_3_N_4_ nanosheets exhibited a uniform thickness of about 3–5 nm and a lateral size of about 1–2 µm. g-C_3_N_4_/graphene nanocomposite was prepared via a self-assembly process. Remarkably, the restacking phenomenon of g-C_3_N_4_ was greatly alleviated after compositing with graphene. A promising sensing performance of g-C_3_N_4_/graphene nanocomposite toward NO_2_ gas at room temperature was demonstrated. The nanocomposite sensor exhibited better recovery as well as two-times the response compared to the pure graphene sensor. The promising performance of the nanocomposite sensor was attributed to a synergetic effect in which the graphene with superior carrier mobility acts as the signal pathway, while the g-C_3_N_4_ nanosheet, with an active surface, plays the role of analyte acceptor.

## Supplementary Materials

The following are available online at http://www.mdpi.com/2079-4991/7/1/12/s1.

## Abbreviations

The following abbreviations are used in this manuscript:

g-C_3_N_4_	Graphitic Carbon Nitride
HCl	Hydrochloric Acid
DI Water	Deionized Water
NO_2_	Nitrogen Dioxide
2D Material	Two Dimensional Material
H_2_SO_4_	Sulphuric Acid
HNO_3_	Nitric Acid
EDS	Energy-dispersive X-ray Spectroscopy
XRD	X-ray Diffraction
FE-SEM	Field Emission Scanning Electron Microscopy
AFM	Atomic Force Microscopy
UV-Vis spectroscopy	Ultraviolet-visible Spectroscopy
MFC	Mass Flow Controllers
