# Supplementary material for: Preparation of g-C3N4/Graphene Composite for Detecting NO2 at Room Temperature
Source: Nanomaterials (Basel). 2017 Jan 12;7(1):12. doi: 10.3390/nano7010012 (PMC5295202; doi:10.3390/nano7010012)
Supplement: Supplementary file 1 [file nanomaterials-07-00012-s001.pdf]

Supplementary Materials: Preparation of g-C_3N_4/Graphene Composite for Detecting NO_2 at Room Temperature

Shaolin Zhang, Nguyen Thuy Hang, Zhijun Zhang, Hongyan Yue and Woochul Yang

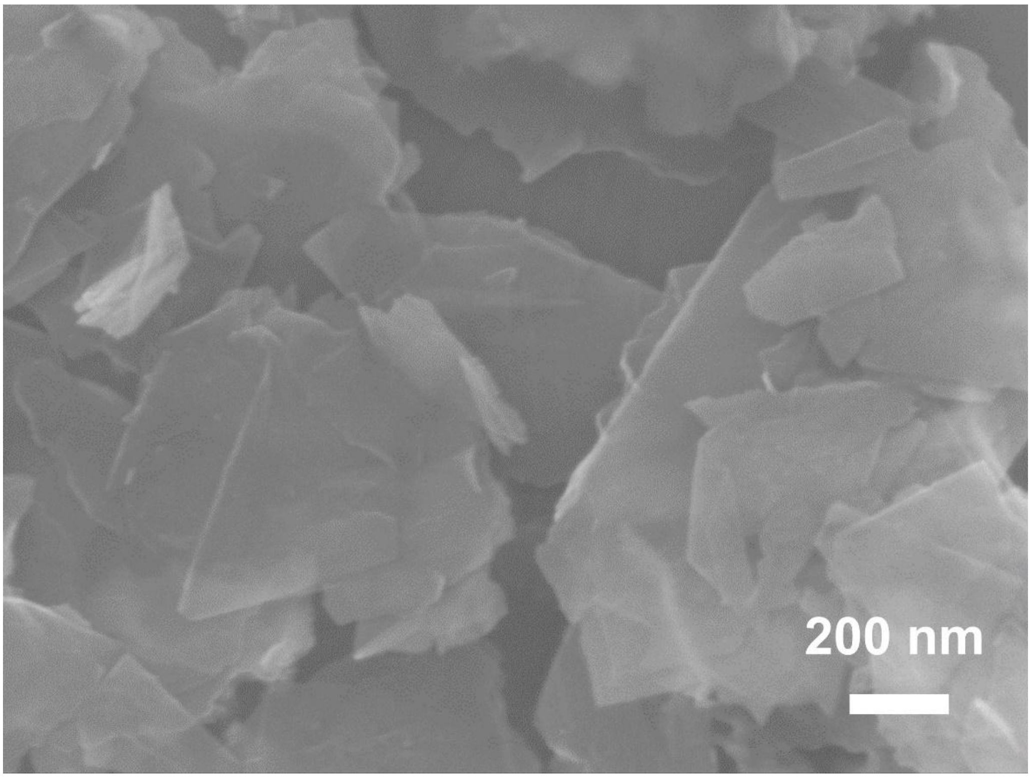

Figure S1. SEM of g-C_3N_4/graphene nanocomposite.

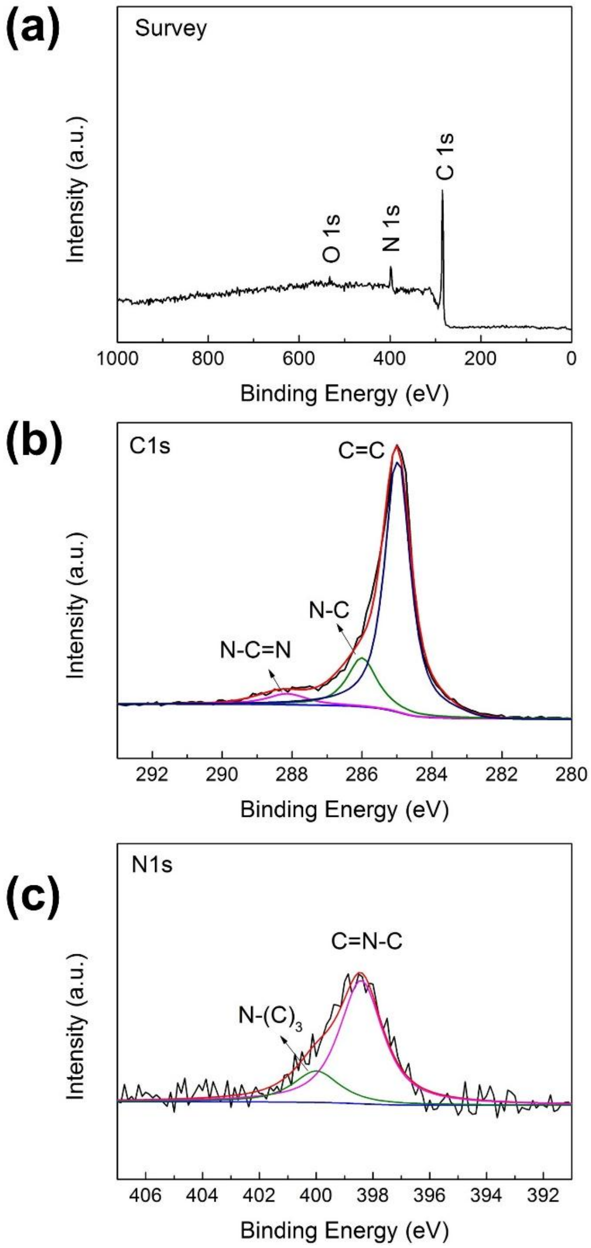

Figure S2. XPS spectra of g-C_3N_4/graphene nanocomposite: (**a**) survey scan; (**b**) C1s; and (**c**) N1s.
